# Oncogene addiction in gliomas: Implications for molecular targeted therapy

**DOI:** 10.1186/1756-9966-30-58

**Published:** 2011-05-17

**Authors:** Wei Yan, Wei Zhang, Tao Jiang

**Affiliations:** 1 Department of Neurosurgery, Beijing Tiantan Hospital, Capital Medical University, No.6 Tiantan Xili, Dongcheng District, Beijing 100050, China

**Keywords:** Oncogene addiction, Glioma, Molecular targeted therapy, Network addiction

## Abstract

Oncogene addiction is a phenomenon that the survival of cancer cells depends on an activated oncogene or inactivation of tumor suppressor gene, and is regarded as the 'Achilles heel' of the successful molecular targeted therapies in cancer. However, the role of oncogene addiction in gliomas has not been elucidated systematically. In this review, we summarize the current experimental and clinical evidence for the concept of oncogene addiction and describe the mechanisms explaining oncogene addiction in gliomas. And the clinical implications for oncogene addiction in molecular targeted therapy are further emphasized. In addition, we discuss future direction for defining complex "oncogene addiction network" through the integrated analysis of multiple platforms in the flow of genetic information in gliomagenesis.

## Introduction

Cancer arises as a result of a stepwise accumulation of genetic aberrations [1]. Despite multiple genetic alterations, its growth and survival can often be impaired by the inactivation of a single oncogene. This phenomenon indicates that tumors may become dependent upon a single oncogenic activity for both maintenance of the malignant phenotype and cell survival [2]. The phrase "oncogene addiction" was coined by Bernard Weinstein to describe the observation that tumor maintenance often depends on the continued activity of certain oncogene or loss of tumor suppressor gene [3]. Oncogene addiction provides a rationale for molecular targeted therapy in cancers [4]. More and more researches proposed that decoding of the oncogene addiction in cancer may provide a key for effective cancer therapy. But it is difficult to define oncogene addiction in numerous conditions. And the efficacy of this strategy requires novel methods, including integrative genomics and systems biology, to identify the status of oncogene addiction in individual cancer [3]. However, it has been known that so many growth related pathways are activated in cancers. To date, it remains controversial whether the cancer cells could get hooked on one single gene [5]. Although the debate that one gene shouldn't affect it much is still continuing, it is remarkable that in some cases reversing only one of these genes can have a strong inhibitory effect. Evidence that supports the concept of oncogene addiction has been obtained in various human cancers via Pubmed Search as indicated in Table 1[6-19].

**Table 1:** Oncogene addiction in various human cancers

Addicted oncogenes	Implications in cancers	Contributors
**MYC**	Inactivation of MYC can result in dramatic and sustained tumor regression in various cancers	**Felsher et al.**, Genes Cancer. (2010) [6]

**cyclin D1**	Cell proliferation	**Lee et al.**, Cell Cycle. (2010) [7]

**Met**	The MET tyrosine kinase stimulates cell scattering, invasion, protection from apoptosis and angiogenesis	**Comoglio et al.**, Nat Rev Drug Discov. (2008) [8]

**PDGFRA amplification or mutation**	Predictive biomarker of drug sensitivity	**Swanton et al.**, Cancer Biol Ther. (2009) [9]

**NF-kappaB**	Acquisition of resistance to CPT	**Togano et al.**, Biochem Biophys Res Commun. (2009) [10]

**FIP1L1-PDGFRalpha**	Generation sustained activation signaling to maintain a cell malignant phenotype	**Jin et al.**, Cancer Sci. (2009) [11]

**PDGF-B**	PDGF-B is required to overcome cell-cell contact inhibition and to confer in vivo infiltrating potential on tumor cells	**Calzolari et al.**, Neoplasia. (2008) [12]

**EGFR amplification or mutations**	Increased sensitivity to EGFR small molecule tyrosine kinase inhibitors	**Rothenberg et al.**, Proc Natl Acad Sci USA. (2008) [13]

**SphK1**	SphK1 is involved in the major mechanisms underpinning oncogenesis	**Vadas et al.**, Biochim Biophys Acta. (2008) [14]

**E2F1**	The E2F1 protein functions as a transcription factor that enhances cell proliferation	**Alonso et al.**, Cancer Lett. (2008) [15]

**HSP90**	Cell proliferation and/or survival	**Workman et al.**, Ann N Y Acad Sci. (2007) [16]

**Bcr-Abl**	Chemosensitivity to imatinib	**Chen et al.**, Cancer Res. (2006) [17]

**mTOR**	mTOR plays a central role in cell growth, proliferation and survival	**Choo et al.**, Cancer Cell. (2006) [18]

**microRNA-21**	Overexpression of miR-21 leads to a pre-B malignant lymphoid-like phenotype	**Medina et al.**, Nature. (2010) [19]

## Oncogene addiction in gliomas

Glioma is the most common primary brain tumor in adults with poor prognosis [20]. The clinical outcomes of patients with glioma traditionally depend upon the tumor pathological grade. But the patients even within the same grade usually have diverse prognosis and therapeutic outcomes [21]. Over the last decade, the knowledge on the molecular genetic background of human gliomas has dramatically increased [22]. However, differences in glioma genetics may result in distinct prognosis and therapeutic outcome, and the underlying mechanism has not been clarified systematically. Underscoring genetic aberrations in gliomas will enhance understanding of tumor biology and have significant clinical relevance for treatment. However, amounts of chromosomal alterations and cancer-causing mutations have been discovered through genome-scale approaches. The complex genetic aberrations provide the basis for molecular targeted therapies, and molecular tests serve to complement the subjective nature of histopathologic criteria and add useful data regarding patient prognosis and therapeutic outcome. Oncogene addiction hides in the above background with complex genetic aberrations. Different types of oncogene addiction can dictate distinct glioma subtypes. It becomes a promising direction to define oncogene addiction for molecular targeted therapy in gliomas. At present, only few oncogene addictions have been identified in gliomas except for E2F1 addiction [15], and some classical glioma-associated genes may be potential oncogene addictions.

EGFR gene amplification or overexpression is a particularly striking feature of glioblastoma (GBM), observed in approximately 40% of tumors. In nearly 50% of tumors with EGFR amplification, a specific EGFR mutant (EGFRvIII) can be detected [23]. This mutant is highly oncogenic and is generated from a deletion of exons 2 to 7 of the EGFR gene, which results in an in-frame deletion of 267 amino acids from the extracellular domain of the receptor. EGFRvIII is unable to bind ligand, and it signals constitutively. Although EGFRvIII has the same signaling domain as the wild-type receptor, it seems to generate a distinct set of downstream signals that may contribute to an increased tumorigenicity [24]. Targeted inhibition of EGFR activity can suppress signal transduction pathways which control tumor cell growth, proliferation, and resistance to apoptosis [25]. Small molecule tyrosine kinase inhibitors and monoclonal antibodies are among the most common EGFR targeting agents and have been used clinically for treating various malignancies [26]. Recently, it was reported that mutations in the tyrosine kinase domain of EGFR gene can predict the response to tyrosine kinase inhibitors [27]. And if alleles with EGFR mutations are amplified, the response to tyrosine kinase inhibitors may differ relative to mutant alleles without gene amplification [28]. Thus, EGFR mutations enable the identification of the glioma subgroup that is likely to be addicted to EGFRs.

Losses of chromosomes 1p and 19q are deemed correlated with the diagnosis of oligodendroglioma, higher PCV chemosensitivity and favorable prognosis [29]. The average rates of 1p deletion and 1p/19q codeletion were respectively 65.4 and 63.3% in oligodendrogliomas, 28.7 and 21.6% in oligoastrocytomas, 13.2 and 7.5% in astrocytomas, 11.6 and 2.9% in glioblastomas [30]. Established indicators of the favorable outcome of oligodendroglial tumors include LOH on chromosomes 1p and 19q, which may indicate a loss of function of as yet unknown tumor-suppressor genes located in those regions [31]. LOH of 1p in the heterogeneous population of malignant gliomas may be one of the vital factors besides MGMT promoter methylation that predict better outcome in patients treated with TMZ [32].

Mutations in IDH1/2 are a common feature of a major subset of primary human brain tumors [33]. Recent studies reported that mutations usually affected amino acid 132 of IDH1 in more than 70% of grade II-III gliomas and secondary glioblastomas. Tumors without mutations in IDH1 often had mutations affecting the analogous amino acid (R172) of the IDH2 gene. Tumors with IDH1 or IDH2 mutations had distinctive genetic and clinical characteristics, and patients with such tumors had a better outcome than those with wild-type IDH genes [34,35]. IDH1 mutation contributes to tumorigenesis partly through induction of the HIF-1 pathway [36]. And it has been recently reported that tumor-derived IDH1 and IDH2 mutations reduced α-KG and accumulated a α-KG antagonist, 2-hydroxyglutarate (2-HG), leading to genome-wide histone and DNA methylation alterations [37]. 2-HG accumulation caused by IDH mutation was also reported to be involved in the formation of malignant gliomas [38]. A recent study has demonstrated that IDH mutation was correlated with a higher rate of response to temozolomide and appeared to be a significant marker of positive prognosis in low-grade gliomas [39]. Taken together, mutations in IDH genes seem to arise from a common glial precursor and play an important role in the formation of specific glioma subtype in which IDH1/2 mutation functions as oncogene addiction.

MicroRNAs (miRNAs) belong to a recently discovered class of small non-coding RNA molecules that regulate the expression of multiple target genes. Some miRNAs, referred to as oncomiRs, show differential expression levels in cancer and are able to affect cellular transformation, carcinogenesis and metastasis, acting either as oncogenes or tumour suppressors. Oncogene addiction to oncomiRs has been proposed in several human cancers [19,40,41]. A lot of studied showed that the aberrant expression miRNAs, including miR-21, miR-221/222, miR-181s and miR-34s, played an important role in gliomagenesis [42-45]. Overexpression of miR-21 could lead to a malignant phenotype, demonstrating that mir-21 was a genuine oncogene. When miR-21 was inactivated, the tumours regressed completely in a few days, partly as a result of apoptosis [42]. And miR-181a and 181b functioned as tumor suppressors in glioma cells [44]. These results demonstrate that tumors could become addicted to oncomiRs and support efforts in treating human cancers through pharmacological inactivation of miRNAs such as miR-21 or upregulation of miR-181s.

## Clinical implications of oncogene addiction in molecular targeted therapy for gliomas

Chemotherapeutic agent therapy or molecular targeted therapy always works in tumors with certain respective genetic background. A growing body of genetic aberrations was identified in gliomas, only a subset of genes acting as drivers in carcinogenesis can be recognized as oncogene addition. Meanwhile, most genes just act as downstream effectors of addicted oncogenes. Oncogene addiction is an ideal potential target for molecular targeted therapy in human cancers. Therapies targeting genes causally linked to carcinogenesis have been successful in a subset of tumor types [46]. Each subtype of gliomas may display a different oncogene addiction. Some molecular targeted drugs only work in a subgroup of tumor patients. The choice of the appropriate molecular targeted agent and combination therapy for a specific patient with cancer is largely empirical. In theory, it is essential to define specific oncogene addiction for individuals before choosing molecular targeted drugs. It should be pointed out that distinct kinds of cells in one sample (e.g. CD133- and CD133+ cells) have different oncogene addictions due to the heterogeneity of glioma. Thus combination of multiple drugs is required to target more than one oncogene addictions in one patient. In addition, oncogene addiction is always moving as the therapeutic targets in gliomas. After exposure to therapeutic agents, cancer cells can escape from one established oncogene addition to another. At this situation, previous drugs would not work anymore. This may be the reason of acquired drug resistance. We named the above phenomenon to "Oncogene addiction transition". Studies are needed for further investigating possible direction of oncogene addiction transition, which is important for choosing rational scheme of combination therapy. Targeting the existing oncogene addiction in combination with blocking the direction of oncogene addiction transition may effectively suppress the growth of glioma cells. Defining oncogene addiction and direction of potential transition in advance based on gene expression profile and bioinformatics analysis will be the novel orientation of combination therapy in the future.

## Approaches for defining oncogene addiction

Recently, the utilities of fluorescence in situ hybridization (FISH), DNA sequencing and methylation specific-polymerase chain reaction (MS-PCR), are widely being employed in assessment of several genetic aberrations for human gliomas [47]. However, it has been reported that systematic characterization of cancer genome has revealed diverse aberrations among different individuals, such that the functional significance and physiological consequence of most genetic alterations remain poorly defined [48]. Cancer cells are characterized by acquired functional capabilities: self-sufficiency in exogenous growth signals, insensitivity to antigrowth signals, limitless replicative potential, evasion of apoptosis, sustained angiogenesis, and acquisition of invasiveness and metastatic ability. The order and mechanistic means to achieve these properties can vary between different tumors. Therefore, cancers are always complex, involving an interplay between various genes and a number of critical pathways and signaling cascades, and the detection of only a single marker molecule is usually insufficient for determining oncogene addiction in gliomas. However, the possibility of developing novel selective drugs against such a large number of genetic aberrations seems extremely daunting. It has been also reported that genetic lesions in cancers tend to cluster around certain pathways, suggesting the concept of 'network addiction', rather than 'oncogene addiction' [46]. It is very difficult to define certain driver genes from amounts of passenger genes in gliomas. Due to the limitation of a single gene or signaling pathway in identifying molecular pattern and predicting clinical prognosis of gliomas, high-throughput screening oncogene addiction networks was highlighted. A lot of single platform analysis cannot identify novel molecular markers that can apply to clinical practice. The integrated analysis of multiple platforms in the flow of genetic information may provide a promising direction for defining oncogene addiction networks. Advances in whole-genome microarray techniques are providing unprecedented opportunities for comprehensive analysis of multi-platform genetic information. The integration of these data sets with genetic aberrations and clinical informations will define novel oncogene addiction networks based on the individual genomics of the patients with glioma. A recent study has showed that a computational approach that integrates chromosomal copy number and gene expression data for detecting aberrations that promote cancer progression [48]. And software has been also developed to identify cancer driver genes in whole-genome sequencing studies [49]. Oncogene addiction networks will likely provide a valuable frame for designing combination therapy of molecular targeted drugs in future.

## Conclusion

Developing novel approaches for defining oncogene addiction networks, coupled with specific combination of molecular targeted agents, will make it possible to achieve more effective and personalized molecular targeted therapy in human gliomas.

## Abbreviations

MiRNAs: MicroRNAs; LOH: Loss of heterozygosity; FISH: fluorescence in situ hybridization; MS-PCR: methylation specific-polymerase chain reaction.

## Author details

^1^Department of Neurosurgery, Beijing Tiantan Hospital, Capital Medical University, No.6 Tiantan Xili, Dongcheng District, Beijing 100050, China.

## Competing interests

The authors declare that they have no competing interests.

## Authors' contributions

TJ initiated the concept. WY and WZ drafted the manuscript. All authors participated in writing, read and approved the final manuscript. WY and WZ contributed equally to this article.
